# Development of generalizable automatic sleep staging using heart rate and movement based on large databases

**DOI:** 10.1007/s13534-023-00288-6

**Published:** 2023-06-08

**Authors:** Joonnyong Lee, Hee Chan Kim, Yu Jin Lee, Saram Lee

**Affiliations:** 1 Mellowing Factory Co. Ltd, Seoul, 06535 South Korea; 2 Department of Biomedical Engineering, Seoul National University College of Medicine, Seoul, 03080 South Korea; 3 Institute of Medical and Biological Engineering, Medical Research Center, Seoul National University, Seoul, 08826 South Korea; 4 Department of Neuropsychiatry, Seoul National University Hospital, Seoul, 03080 South Korea; 5 Transdisciplinary Department of Medicine and Advanced Technology, Seoul National University Hospital, Seoul, 03080 South Korea; 6 Center for Sleep and Chronobiology, Seoul National University Hospital, Seoul, 03080 South Korea

**Keywords:** Automatic sleep stage scoring, Home sleep monitoring, Polysomnography, Heart rate, Deep neural networks

## Abstract

**Purpose:**

With the advancement of deep neural networks in biosignals processing, the performance of automatic sleep staging algorithms has improved significantly. However, sleep staging using only non-electroencephalogram features has not been as successful, especially following the current American Association of Sleep Medicine (AASM) standards. This study presents a fine-tuning based approach to widely generalizable automatic sleep staging using heart rate and movement features trained and validated on large databases of polysomnography.

**Methods:**

A deep neural network is used to predict sleep stages using heart rate and movement features. The model is optimized on a dataset of 8731 nights of polysomnography recordings labeled using the Rechtschaffen & Kales scoring system, and fine-tuned to a smaller dataset of 1641 AASM-labeled recordings. The model prior to and after fine-tuning is validated on two AASM-labeled external datasets totaling 1183 recordings. In order to measure the performance of the model, the output of the optimized model is compared to reference expert-labeled sleep stages using accuracy and Cohen’s κ as key metrics.

**Results:**

The fine-tuned model showed accuracy of 76.6% with Cohen’s κ of 0.606 in one of the external validation datasets, outperforming a previously reported result, and showed accuracy of 81.0% with Cohen’s κ of 0.673 in another external validation dataset.

**Conclusion:**

These results indicate that the proposed model is generalizable and effective in predicting sleep stages using features which can be extracted from non-contact sleep monitors. This holds valuable implications for future development of home sleep evaluation systems.

## Introduction

With mounting evidence relating sleep deprivation to chronic health issues such as diabetes [1–4], stroke [5], depression [6] and Alzheimer’s disease [7], the importance of sleep has become clearer recently [8]. While the combination of these insights and the high prevalence of sleep disorders [8–11] warrant attention, sleep disorders are often left undiagnosed due to the difficulties in testing [12]. Currently, the standard method to diagnose sleep disorders is through a polysomnography (PSG) study, which requires an overnight measurement of biosignals including electroencephalogram (EEG), electromyogram (EMG), and electrooculogram (EOG). Following the measurement session, the record must be manually analyzed by a trained expert following heuristic sleep scoring standards, making the task resource-intensive. Naturally, to reduce labor costs, methods to automate sleep scoring have been proposed since the inception of PSG [13].

Sleep scoring task presents a classical machine learning problem which requires extracting information from multi-dimensional sequence data in order to predict the state of a particular system. In the past decade, the development of deep learning methods and large-scale PSG data has allowed for deep neural network based methods to thrive in this task, with reported accuracies reporting up to 99% in 8 validation recordings [14], up to 87.5% in 580 validation recordings [15], and various results in between [16–20]. Although some of these methods are accurate enough to be deployed in commercial PSG systems as an alternative to expert labeling, a PSG session is still limited to the clinic due to the measurement of various biosignals. Problematically, even when a patient undergoes a PSG session at the clinic, the measurement conditions with sensors attached to the subject may disturb regular sleep [21], and the results may be unreliable or uncharacteristic of their regular sleep cycle. These limitations make repeat studies of PSG difficult, and thus there has been a push to develop sleep scoring methods based on unobtrusive signals which could be used in home settings.

As expert labeling of sleep cycles depends heavily on EEG and EOG, sleep scoring models are much less accurate without the use of these biosignals. Previous works have attempted to use only commonly available biosignals such as electrocardiogram (ECG) and photoplethysmogram (PPG) for sleep scoring [22–24] but with small homogeneous subject pools and without external validation on a separate dataset. A study in 2020 [25] has used a larger dataset consisting of about 10,000 recordings derived from three publicly available databases, but the results lacked relative accuracy in distinguishing between NREM (Non-REM, rapid eye movement) sleep cycles, with about 50% of N3 stage being predicted as N1/N2 stages. As the authors state, one potential reason for this inaccuracy of N3 detection may be due to the fact that the training data and the external validation data were labeled using different standards. The majority of publicly available PSG data as of this study are labeled using the Rechtschaffen & Kales (R&K) scoring system [26], while the current gold standard for sleep scoring follows the American Association of Sleep Medicine (AASM) guidelines [27]. A study in 2009 of 72 subjects found significant differences in sleep characteristics when the same PSG record was labeled using the two standards, with increased slow wave sleep (S3 + S4 vs N3, + 9.1 min), increased stage 1 sleep (S1 vs N1, + 10.6 min), decreased stage 2 sleep (S2 vs N2, − 20.5 min), and increased wake after sleep onset (approximately 4 min) for the scoring following the AASM guidelines [28]. These differences imply challenges in developing automatic sleep staging models using the currently available data to match the current gold standard for sleep scoring.

In this study, we propose a deep neural network model for sleep staging using heart rate and movement. The model was first trained using a large set of R&K-labeled data, then fine-tuned using a smaller dataset labeled with the AASM standard. In the next section, the model architecture and the databases used to train the model are introduced. Then, the predictions of the optimized model with and without fine-tuning are compared in the external AASM validation datasets. The results are discussed in relation to model architecture, sleep characteristics of the datasets, and to the scoring standards. Lastly, the presented methodology is discussed in terms of applicability to home-use.

## Materials and methods

### Datasets and data exclusion process

Six databases were used in this study, the Sleep Heart Health Study (SHHS) [29] and the Wisconsin Sleep Cohort (WSC) [30] were used for the initial training of the model based on the R&K scoring. The Multi-Ethnic Study of Atherosclerosis (MESA) [31] database, the Mignot Nature Communications (MNC) database [32], the NCH Sleep Databank (NCHSDB) database [33] were used to fine-tune the model according to AASM scoring. Lastly, an internal database generated at the Seoul National University Hospital (SNUH) (IRB no. 2101–116-1190) and 2018 Computing in Cardiology Conference (CCC) [34] databases were used for the external validation of the model.

SHHS, WSC, MESA, MNC, and NCHSDB are publicly available from National Sleep Research Resource [35] and the CCC is available through Physionet [36]. All datasets were expert-labeled using following the R&K or the AASM scoring guidelines, and included patients with various sleeping disorders. For direct comparison to previous works, epochs labeled as S1, S2, N1, N2 were relabeled to ‘light sleep’, and epochs labeled S3, S4, N3 stages were relabeled to ‘deep sleep’, while stages labeled as ‘wake’ and ‘REM’ were retained.

**Fig. 1:**
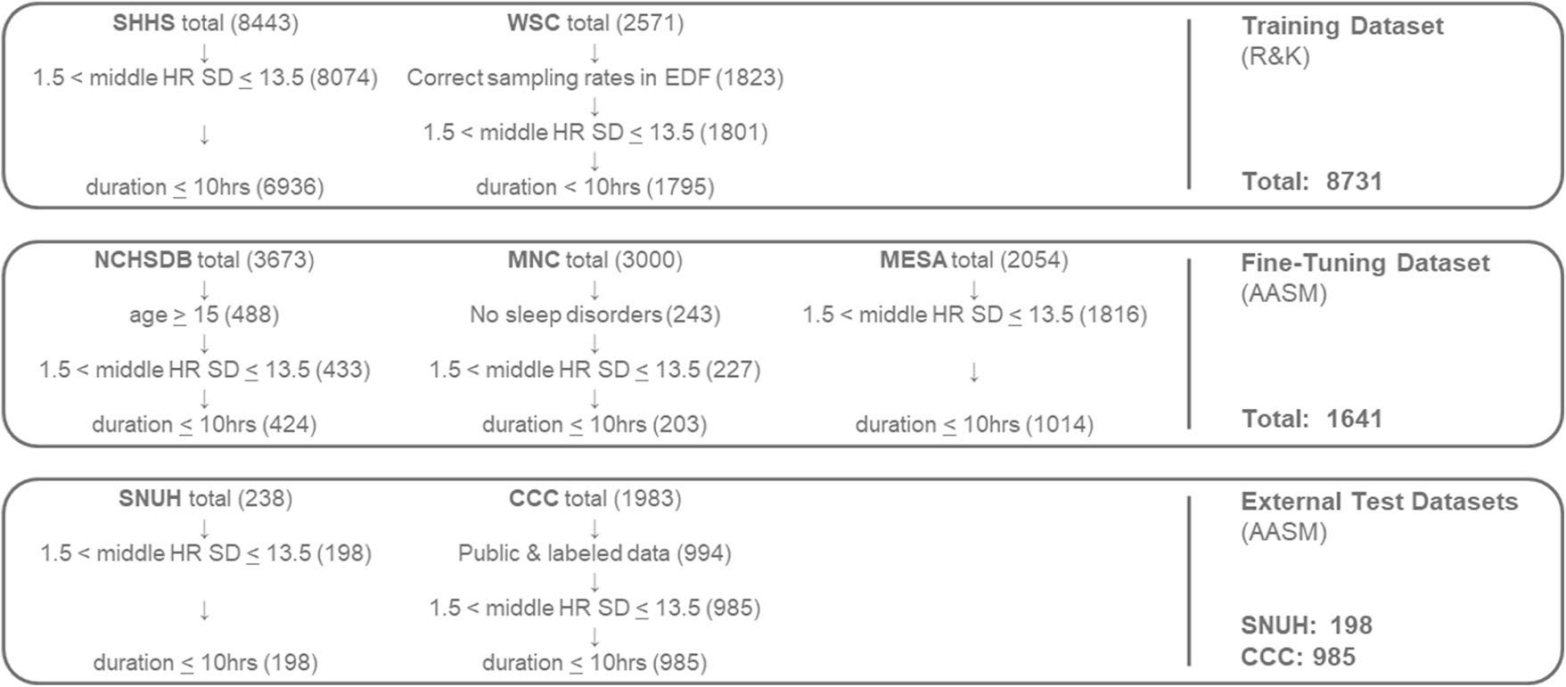
Data exclusion process for each dataset used in this study. Middle HR SD indicates standard deviation of heart rate in the middle 50% duration of the recording. AASM, American Association of Sleep Medicine; R&K, Rechtschaffen & Kales; ECG, electrocardiogram; ABD, abdominal excursion; HR, heart rate; SD, standard deviation; EDF, European data format file; Acronyms for database names can be found in the text

From all datasets, recordings longer than 10 h were excluded and recordings without ECG or abdominal excursion signal were excluded. Additionally, following the extraction of heart rate from ECG signal (Sect. 2.2), recordings were excluded if the standard deviation of the heart rate was below 1.5 BPM or above 13.5 BPM in the middle 50% duration of the recording in order to remove data with large heart rate detection errors. For the WSC dataset, recordings with ECG sampling rates greater than 1000 were excluded, since the total duration of ECG did not match the total duration of sleep stage labels in these recordings. For the MNC database, only subjects without any sleeping disorders were included, as this data was intended for the study of narcolepsy. For the NCHSDB dataset, subjects below 14 years of age were excluded. This age limit was set following the AASM guidelines, which states that PSG studies for children above the age of 13 years may be scored following the criteria for adult subjects [27]. For the CCC database, the recordings with labeled sleep stages were included. In total 8731 recordings labeled with the R&K standard were used for the initial training of the model, 1641 recordings labeled with the AASM standard were used to fine-tune the model, and two separate datasets of 198 and 985 recordings were used for external validation of the models following initial optimization and after fine-tuning (Fig. 1). Although some databases were longitudinal with multiple PSG recordings from some subjects, these recordings were used as independent samples.

### Prediction features and sleep stage labels

Data processing and feature extraction were performed using Python3 and open-source libraries. In this study, only unobtrusive features (i.e. features that can be easily extracted from non-contact sensors) which could be derived from PSG were considered, while disregarding any features that require contact sensors such as EEG or EOG. In this regard, heart rate and movement were considered for sleep staging, since these could be extracted from signals measured in non-contact home sleep monitors. However, because these non-contact signals were not available in the open PSG databases, they were extracted from the available signals including ECG and abdominal excursion. As the variations in sympathetic and parasympathetic tone during each sleep stage causes changes in heart rate [37–40], features directly related to heart rate were extracted in 15-s intervals. Additionally, a feature representing the movement of the user was extracted in the same intervals. As the ranges for these features varied widely for each subject due to variations in physiology and the mode of measurement, features were standardized for each recording by subtracting the mean across the recording then dividing by the standard deviation values (Eq. 1).

1 $$X_{standardized} = \frac{{X - \mu_{X} }}{{\sigma_{X} }}$$

**Fig. 2:**
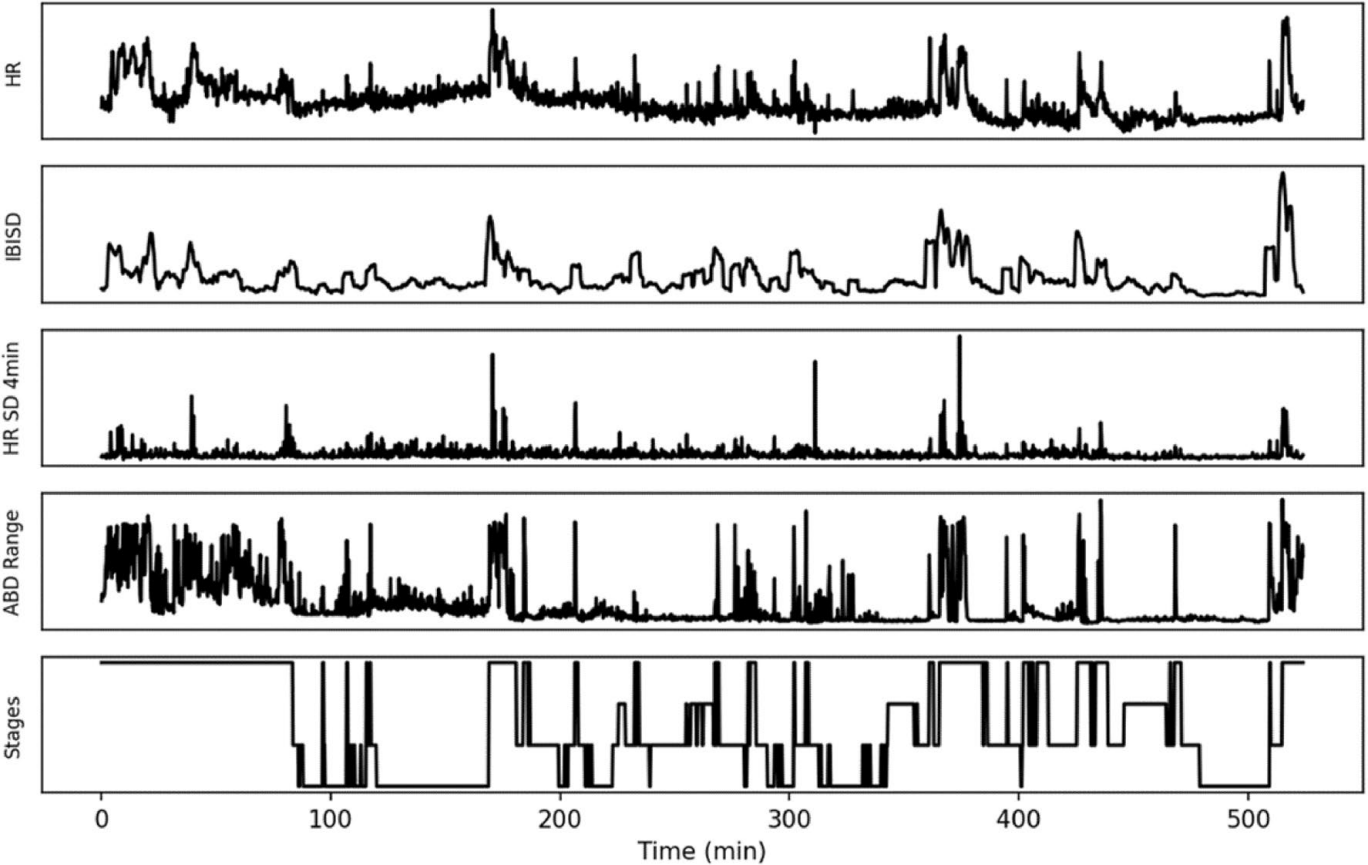
Features used in this study extracted from subject 200003 in the SHHS1 dataset. Sleep stages are shown upside-down to reflect conventional hypnogram plotting. HR, heart rate; IBI, inter-beat-interval; SD, standard deviation; ABD, abdominal excursion

Regions of the recording where certain features couldn’t be found were linearly interpolated using the Pandas library [41] with maximum limit of 4 samples for interpolation. A sample of the features and sleep stages is shown on Fig. 2.

Inter-beat interval (IBI) between consecutive ECG R-peaks in 15-s windows were extracted using the BioSPPy library [42]. Average heart rate was calculated from the IBIs in the 15-s window, and the standard deviation of the IBI (IBISD) was calculated as a representation of short-term heart rate variation. For every 4-min window, standard deviation was calculated to represent longer-term heart rate variation (4-min HR SD).

Signal range of the abdominal excursion signal was used to represent movement, as this signal was the closest to the conventional signal measured from non-contact home sleep monitoring devices and because large bodily movements would cause saturation in the signal, by subtracting the minimum of the abdominal signal from the maximum of the signal in 15-s interval (ABD range).

Labels for the 15-s intervals were extracted from the 30-s epoch labels by copying the epoch label values in the corresponding 15-s interval. Labels that were unknown were interpolated using adjacent labels, and when the labels were unknown in the beginning or the end of the recording, these were re-labeled as ‘wake’. For training and validating the model, ‘wake’ was defined as 0, ‘REM’ was defined as 1, ‘light sleep’ was defined as 2, and ‘deep sleep’ was defined as 3.

### Deep neural network architecture

**Fig. 3:**
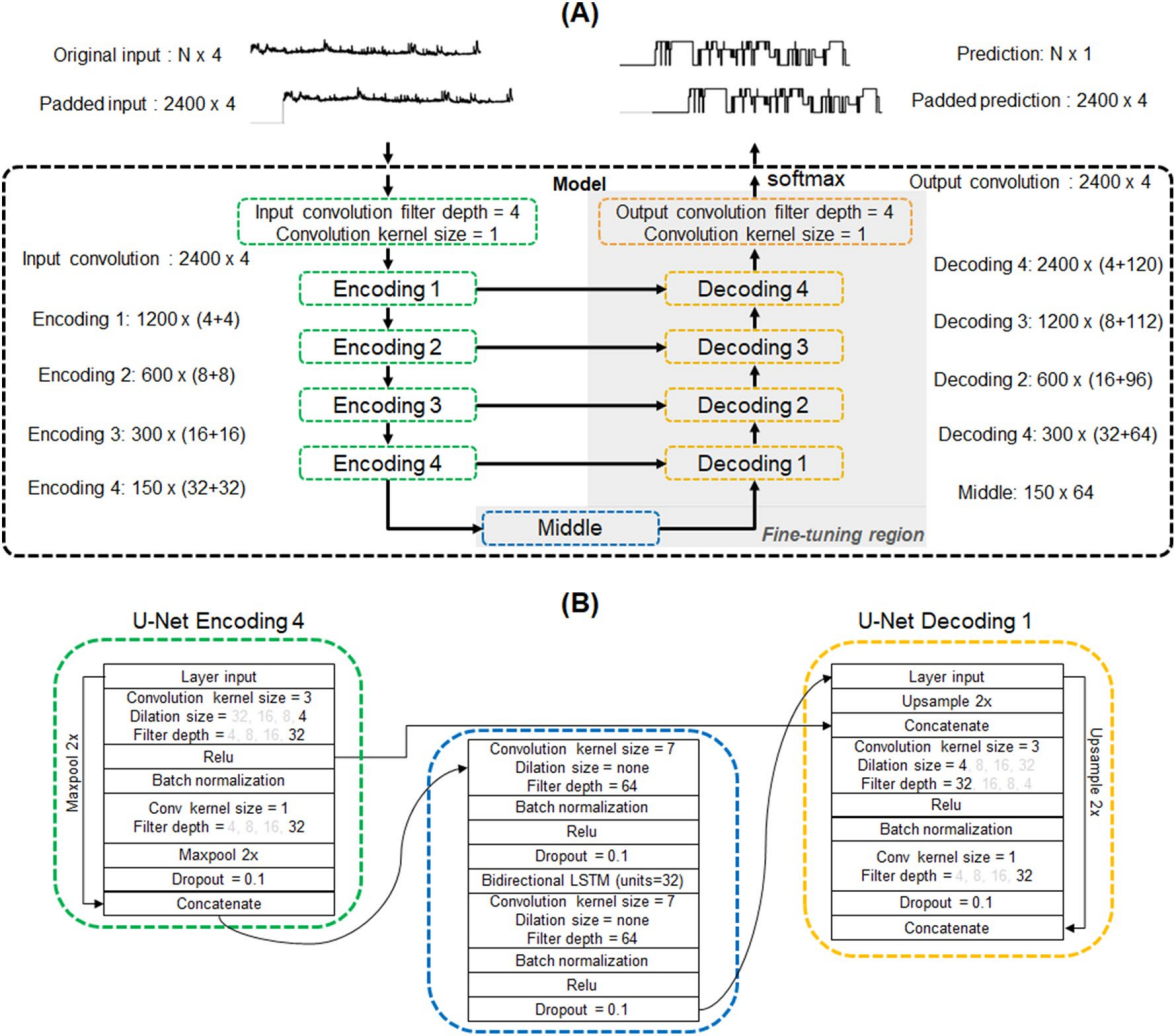
Proposed model architecture for automatic sleep staging. Only 1 input feature is shown at the input layer for clarity. **A** Overall architecture of the model showing the input, encoding layers, middle layers, decoding layers, and prediction with respective shapes at the output of each layer. Addition shown in parenthesis for each shape value represents concatenation of residual outputs in a given block. Gray-shaded region indicates the layers of the network that were fine-tuned. **B** View of the middle part of the model, with details of encoding layer 4, middle layers with recurrent architecture, and decoding layer 1. The grayed-out values on dilation size and filter depth represents the values for the other layers in the encoding or decoding blocks. Relu, rectified linear unit; LSTM, long short term memory

The details of the deep neural network architecture used in this study can be seen on Fig. 3. The input to the network is composed of 2400 sequential features (2400 × 4) including IBI, IBISD, 4-min HR SD, and ABD range measured over 10 h of sleep. If the duration of a PSG record did not reach 10 h, the features and the labels were padded at the beginning with zeros to match 10 h. The network has a U-Net [43] shape with features being encoded with 4 layers of dilated convolutions, middle block with convolution and recurrent layers, decoding with 4 layers of dilated convolutions, and residual connections between the encoding and decoding layers. Lastly, the decoded output is activated with softmax to represent the probability of each class in the output 4-class hypnogram. In total, the model consisted of 76,552 trainable parameters and 496 non-trainable parameters. The output of the model was compared to the reference hypnogram using cross-entropy.

### Model training, validation, and fine-tuning

The training hyperparameters were chosen based on experimental search. A batch size of 100 was used for all training steps. Following each training, the model was tested on the external validation datasets to calculate the corresponding accuracy, Cohen’s κ, and confusion matrix.

#### Initial optimization with R&K-labeled dataset

For the initial training, an Adam optimizer with initial learning rate of 1e-4 was used to update the model parameters according to cross-entropy loss. The data from the R&K dataset were randomly mixed and partitioned in 4-to-1 ratio for training and validation, resulting in 6985 recordings for training and 1746 recordings for internal validation.

#### Fine-tuning with AASM-labeled data

To optimize the model to the AASM scoring standards, the model from the above was fine-tuned using the AASM-labeled dataset. In the same manner, the data was mixed and partitioned for training and validation, resulting in 1313 recordings for training and 328 recordings for internal validation. The weights of the encoding layers of the model were kept frozen, and only the middle and the decoding layers were updated using an Adam optimizer with initial learning rate of 1e-5.

#### AASM-model training without fine-tuning

To compare the results of the model on AASM-labeled data with and without fine-tuning using the R&K-labeled data, the same model was trained solely on AASM-labeled data with randomly initialized layers. An Adam optimizer with initial learning rate of 1e-4 was used to update the model parameters.

## Results

**Fig. 4:**
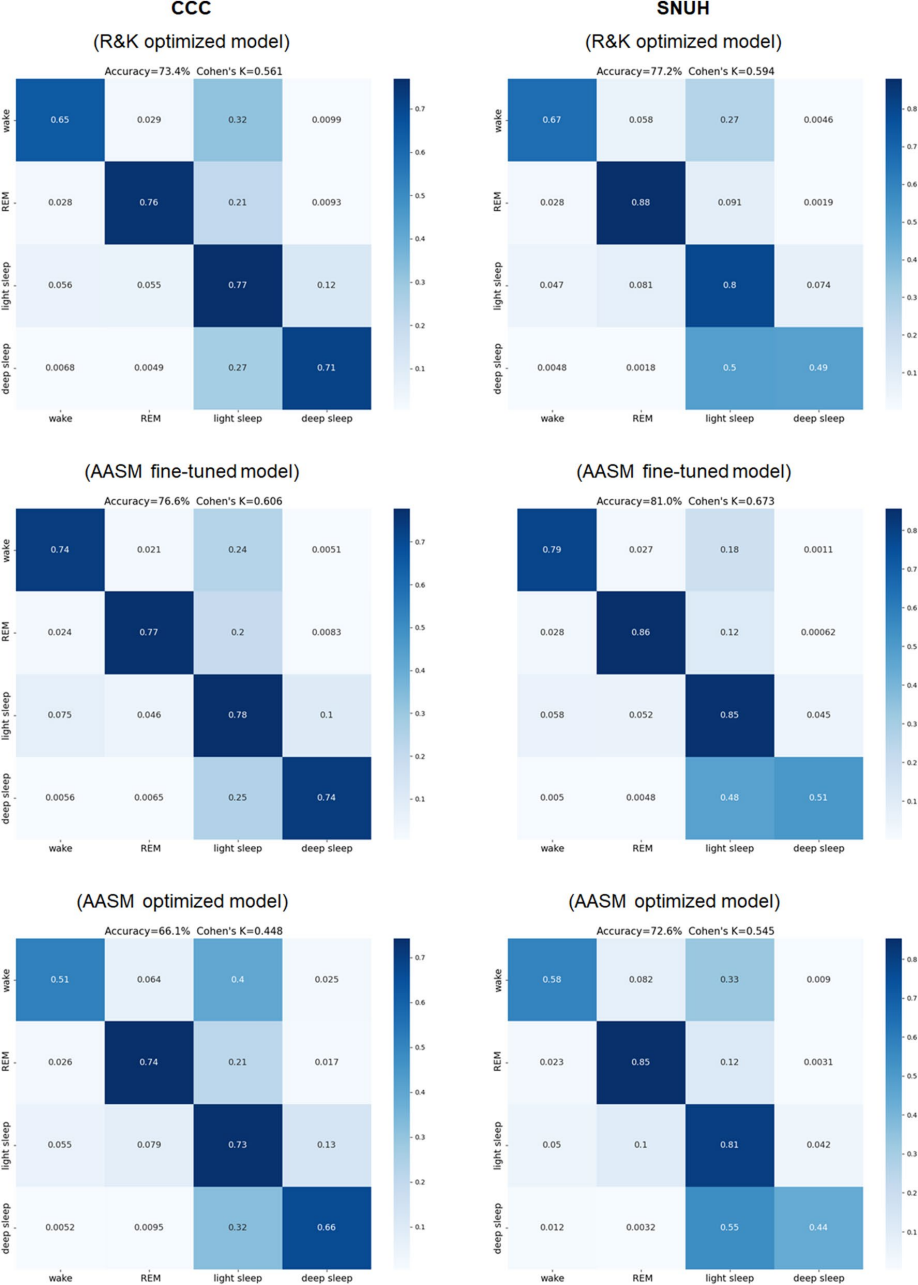
Confusion matrices calculated for the model at each step of optimization on the two external validation datasets. The top row corresponds to results of the prediction model solely trained on R&K-labeled datasets. The middle row corresponds to the results of the model following fine-tuning using AASM-labeled datasets. The bottom row corresponds to the results of the model solely trained on AASM-labeled datasets. AASM, American Association of Sleep Medicine; R&K, Rechtschaffen & Kales

Following R&K optimization, prediction on CCC resulted in accuracy of 73.4% with Cohen’s κ of 0.561 and accuracy of 77.2% and Cohen’s κ of 0.594 for SNUH. After fine-tuning using AASM-labeled datasets, prediction on CCC resulted in accuracy of 76.6% with Cohen’s κ of 0.606 and accuracy of 81.0% and Cohen’s κ of 0.673 for SNUH. For the model solely trained on AASM-labeled datasets, CCC prediction led to accuracy of 66.1% and Cohen’s κ of 0.448, while SNUH prediction showed accuracy of 72.6% and Cohen’s κ of 0.545. These results are summarized in the corresponding confusion matrices shown in Fig. 4

**Fig. 5:**
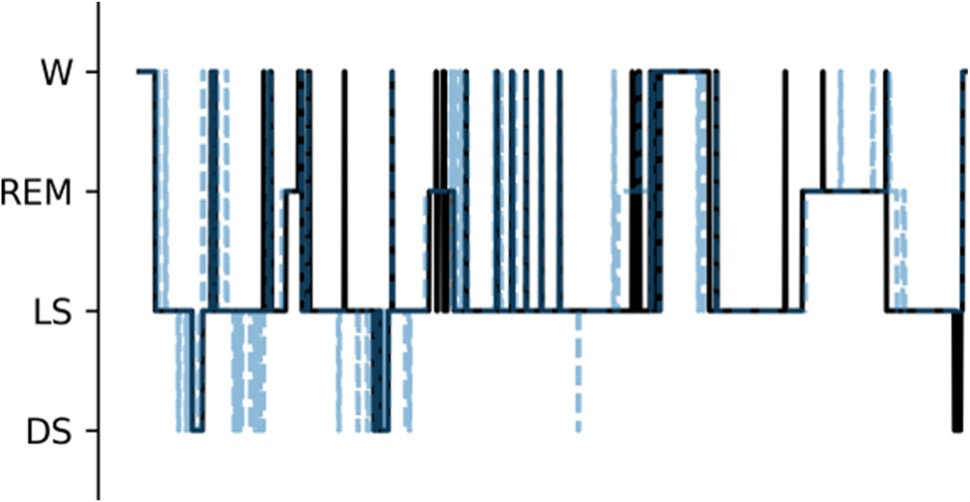
Example of a predicted hypnogram following fine-tuning plotted in solid lines and the corresponding reference hypnogram in dotted lines for the SNUH dataset with Cohen’s κ of 0.66 and accuracy of 83%. The results are in line with the confusion matrix, showing lowest prediction accuracy for the deep sleep stage. W, wake; REM, rapid-eye movement; LS, light sleep; DS, deep sleep

A median example of the predicted hypnogram and the reference hypnogram are shown in Fig. 5, and performance comparison against a previous study is presented on Table 1.

**Table 1:** Comparison of external validation results to a prior study

Author (database)	Recordings	Accuracy (%)	Cohen’s κ
Sridhar et al*. *[25] (CCC)	993	72.0	0.550
This study (CCC)	985	76.6	0.606
This study (SNUH)	198	81.0	0.673

## Discussion

The method proposed in this study yielded significant results in sleep staging using unobtrusive features which could be measured easily in an out-of-clinic setting. Due to the nature of deep neural network models to overfit the training data, thorough external validation is critical for developing a widely generalizable sleep staging model which can be applied to different sleep patterns. As seen on Table 1, the external validation of the fine-tuned model led to overall improved results in the CCC database and in the SNUH database. However, the results were not in-line with the initial hypothesis that fine-tuning using AASM-labeled dataset will lead to better prediction in deep sleep stage, and this will be discussed in Sect. 4.2.

### Model architecture, feature selection, and performance

The proposed model architecture could be understood as having three distinguished parts composing a U-Net like architecture. The encoding part, formed of 4 encoding blocks, extracts relevant information from the input features for sleep staging. The middle block learns the sequential patterns from the output of the encoding block and inputs to the result into the decoding block. Lastly, the decoding block converts the features maps into a 4-class output hypnogram.

Various architectures were attempted in this study, but removing any of the major blocks in the architecture (encoding block, middle block, decoding block) resulted in decreased accuracy, adding layers or nodes to these blocks did not improve the outcome, and increasing the number of trainable parameters in each block also did not improve the performance. In regards to input size, 15-s features were used to predict 15-s labels, as this experimentally outperformed 30-s features predicting 30-s epoch labels. The performance increased with decreasing window size, but the window was set at 15 s for this study, as to limit the input and output sizes small enough such that training could be done on the available workstation.

Initially, different types of features were extracted, including breathing rate, but the model performance was not improved, perhaps due to the low accuracy of detection using zero-crossings as an indicator of a breath cycle in the abdominal excursion signal. Different types of heart rate related features and movement related features were also considered, but did not change the model performance. Such features included low and high frequency spectral features of heart rate and features such as entropy, kurtosis, and skewness related to movement as reflected in the abdominal signal. Hence, it seems likely that any further modification in the network architecture without the addition of meaningful prediction features will not result in further improvement in the prediction performance. Future studies should be focused on discovering additional features for sleep staging that are uncorrelated with the features used in the current study, perhaps using alternative sensor modalities. In line with the goal of unobtrusive sleep staging outside the clinic, potential feature candidates include sound [44] and actigraphy [45].

### Fine-tuning and database characteristics

The key hypothesis of this study was to check if fine-tuning could lead to improved performance with an increased detection of deep sleep in AASM-labeled data. Although the overall performances improved, the accuracy in deep sleep prediction did not increase dramatically in the external validation datasets, and in the case of SNUH, the detection of REM stage was slightly worse following fine-tuning. The biggest improvement in accuracy was seen in the detection of wake stages, with less predictions being mislabeled as light sleep. As seen, training the model with AASM data alone led to decreased model performance, possibly due to the low number of data available. Additionally, optimizing the model without fine-tuning, with all R&K and AASM-labeled data together also resulted in similar performance as the model trained using just the R&K-labeled dataset. Fine-tuning all of the model including the encoding region or fine-tuning just the decoding region also did not improve model accuracy. These results together indicate that the final performance of the sleep staging model may be dependent on the underlying characteristics of the training data applied at different levels of optimization.

As seen on Table 2, each dataset used in this study had different sleep characteristics. The external validation datasets had lower percentages of wake stages as compared to the initial training and the fine-tuning datasets, and improved in wake stage prediction performance after fine-tuning. The fine-tuning dataset had a higher percentage of wake stages as compared to the initial training dataset, and thus it could be hypothesized that additional data with more wake stages, along with the fact that AASM guidelines label more stages as wake during sleep onset as compared to R&K, led to a better generalization of this stage in external validation. Furthermore, the fine-tuning dataset had a lower percentage of deep sleep stages as compared to the initial training dataset, and the improvement in deep sleep prediction performance was only slight in the external validation following fine-tuning – which may be due to the fact that the R&K standard is different to AASM standard in terms of labeling deep sleep, and the model was fine-tuned to match AASM scoring. Therefore, additional data for fine-tuning with greater distribution of a particular stage may lead to better prediction performance.

**Table 2:** Sleep characteristics of the datasets

	Initial training	Fine-tuning	External validation	
			CCC	SNUH
Standard	R&K	AASM	AASM	AASM
Recordings	8731	1641	985	198
Duration (Epoch)	1014 ± 92	1085 ± 113	925 ± 79	948 ± 76
Efficiency (%)	71 ± 12	65 ± 16	80 ± 13	85 ± 11
Wake (%)	29 ± 12	33 ± 16	17 ± 13	15 ± 11
REM (%)	12 ± 14	12 ± 6	13 ± 7	18 ± 6
Light sleep (%)	47 ± 12	45 ± 12	56 ± 13	60 ± 10
Deep sleep (%)	10 ± 8	8 ± 8	11 ± 8	7 ± 7

Kruskal–Wallis H-test between sleep characteristics of the datasets all yielded significant *p*-values. Efficiency plus wake percentage may not add to 100% due to presence of unlabeled epochs, averaging, and rounding errors.

The results seen above were not in-line with our initial hypothesis that fine-tuning on AASM dataset will lead to significant improvements in deep sleep prediction during external validation. More than the differences in scoring standards of deep sleep stage for the R&K system and the AASM system, the differences in the distribution of stages for the various datasets seem to affect the final external validation performance. However, as training with the combined R&K and AASM datasets led to worse results than the proposed method, there may also be different factors at play which has to do with the order in which the training occurs. By training the model first with a larger R&K dataset to achieve generalizability in encoding information from the input features, and fine-tuning the sequential prediction and decoding with the AASM dataset, which had a larger distribution of wake stages, the model weights may have adjusted to better predict wake while keeping the generalizability of encoding the input features. Although this idea was further tested by stratifying the AASM fine-tuning dataset to have increased distributions of each stage, it could not be recreated for the other stages, as increasing the distribution of one stage led to decreasing the distribution of the other stages. Hence, a further study is warranted using larger datasets to determine the effect of sleep characteristics on the performance of proposed fine-tuning method.

### Future work towards an unobtrusive solution for home sleep scoring

Evidence relating sleep disorders with many comorbidities is mounting, yet testing of sleep disorders remains low due to the difficulties of PSG, and thus patients frequently remain undiagnosed. A low cost, home-based solution may be able to provide wide-spread and frequent testing of sleep disorders. Much research about automatic sleep staging has been focused on using a wide range of biosignals, but solutions that require professional equipment such as EEG and ECG are not easily applicable for home-use. In the recent decade, there has been an explosion of wide variety of home sleep monitoring solutions. Products ranging from smartwatches to smartphone applications offer unobtrusive monitoring of various physiological signs in order to predict the user’s sleep pattern. Sleep scoring at home has the benefit of measuring regular sleep without the stress of sleeping at a clinic, as well as repeatability of multiple measurements to yield an average sleep pattern of the user. The proposed methodology demonstrates that a deep neural network model can be used to detect sleep stages with high accuracy using heart rate and movement as input features across a wide range of subjects, and that fine-tuning can be used to overcome the lack of AASM-labeled data and improve the model performance for the current standard of sleep scoring. The utility of such algorithm with limited performance as compared to the gold standard must be answered by clinicians with sufficient evidence. In order to get to this next stage, as discussed above, there are issues that must be addressed. First, the model must be retrained with 5-stage hypnogram as the target output. Although this was attempted, the ability of the model to distinguish N1 from N2 stage was poor, likely due to the low durations of the N1 stage in most recordings. Second, various sleep disorders may have different effects on prediction performance, and must be taken into account when applying the model to a new subject. A potential solution for this issue would be to further optimize the model into recognizing various disorders, for example by adding breathing events as input features to adjust the final prediction for sleep apnea patients.

### Limitations

There are some limitations to the proposed method. The first limitation of the model has to do with its unpredictable nature as a black box model. It may be difficult to improve the model performance for particular stages as desired without flooding the model with additional data to train on. In this regard, automatic sleep staging using deep learning faces a data imbalance problem; there is only a limited number of available data, and the distribution of this data and the sleep characteristics are often irregular. As seen in Fig. 4, the proposed model tends to overestimate light sleep, and this is likely due to the fact that each recording on average has 50% of stages labeled as light sleep. In an ideal problem, the distribution of sleep stages is fairly even such that the model is not biased towards a particular stage. A better approach to this issue may be to balance out the number samples based on the labeled sleep stage. However, the balancing of the samples requires additional research for even distribution of sleep stages, and changes in sleep stages. This is beyond the scope of this study, and may be better accomplished in the future when more data is readily available for such research.
